# Commentary: Open and Laparoscopic Colposuspension in Girls With Refractory Urinary Incontinence

**DOI:** 10.3389/fped.2018.00067

**Published:** 2018-03-27

**Authors:** Miguel Podesta, Ricardo González

**Affiliations:** ^1^Hospital General de Niños Ricardo Gutierrez, Buenos Aires, Argentina; ^2^Facultad de Medicina, Universidad de Buenos Aires, Buenos Aires, Argentina; ^3^Pediatric Surgery and Urology, Kinder- und Jugendkrankenhaus AUF DER BULT, Hanover, Germany; ^4^Urology, Charité Universitätsmedizin Berlin, Berlin, Germany

**Keywords:** urinary incontinence, bladder neck insufficiency, colposuspension, adolescent, laparoscopic surgery, Burch procedure

This is a nice study where the authors have tried to demonstrate the comparative efficacy of open and laparoscopic colposuspension in girls with refractory urinary incontinence.

However, this retrospective study is limited by an incompletely defined patient population, since all patients who underwent colposuspension had refractory urinary incontinence with an open bladder neck during filling, hypermobile bladder neck and a flat vesico-urethral angle.

As the authors point out, simultaneous video-pressure-flow cystorethrography is of great value to accurately diagnose the cause of refractory urinary incontinence. However, it is not clear in the paper how many patients had an anatomic malposition of the bladder neck, how many had intrinsic sphincter deficiency and how many had both. This distinction is important since the ideal surgical options for intrinsic sphincter deficiency, are injection of periurethral bulking agents, sling procedures or artificial sphincter placement. Colposuspension only makes sense when the bladder neck is hypermobile, a situation rarely seen in nulliparous adolescent females, but not in congenital bladder neck insufficiency.

Detrusor hyperactivity resulting from an open bladder neck during the filling phase has not been described and we have personally never observed it. A final commentary is that the success rate of a surgical procedure for incontinence treatment should not be based on clinical evidence alone but on objective evaluation test.

Given the rather poor results in the reported series, the reader would be well advised to use colposuspension (open or laparoscopic) in this patient population with caution and only after solid documentation of bladder neck hypermobility.

## Author’s Note

This is a commentary on an article included in the Research Topic: “Urinary Incontinence in Children: Controversies Concerning the Bladder Outlet”.

## Author Contributions

Both authors contributed equally to the conception and writing of this commentary.

## Conflict of Interest Statement

The authors declare that the research was conducted in the absence of any commercial or financial relationships that could be construed as a potential conflict of interest.

